# A Single Prior Injection of Methamphetamine Enhances Methamphetamine Self-Administration (SA) and Blocks SA-Induced Changes in DNA Methylation and mRNA Expression of Potassium Channels in the Rat Nucleus Accumbens

**DOI:** 10.1007/s12035-019-01830-3

**Published:** 2019-11-22

**Authors:** Subramaniam Jayanthi, Oscar V. Torres, Bruce Ladenheim, Jean Lud Cadet

**Affiliations:** 1 Molecular Neuropsychiatry Research Branch, Intramural Research Program, NIDA/NIH/DHHS, 251 Bayview Boulevard, Baltimore, MD 21224 USA; 2 Department of Behavioral Sciences, San Diego Mesa College, San Diego, CA USA

**Keywords:** Addiction, DNA methylation, Potassium channels, Substance use disorder (SUD)

## Abstract

The transition from occasional to escalated psychostimulant use is accelerated by prior drug exposure. These behavioral observations may be related to long-lasting transcriptional and/or epigenetic changes induced by the drug pre-exposure. Herein, we investigated if a single methamphetamine (METH) injection would enhance METH self-administration (SA) and impact any METH SA-induced epigenetic or transcriptional alterations. We thus injected a single METH dose (10 mg/kg) or saline to rats before training them to self-administer METH or saline. There were three experimental groups in SA experiments: (1) a single saline injection followed by saline SA (SS); (2) a single saline injection followed by METH SA (SM); and (3) a single METH injection followed by METH SA (MM). METH-pretreated rats escalated METH SA earlier and took more METH than saline-pretreated animals. Both groups showed similar incubation of cue-induced METH craving. Because compulsive METH takers and METH-abstinent rats show differences in potassium (K^+^) channel mRNA levels in their nucleus accumbens (NAc), we wondered if K^+^ channel expression might also help to distinguish between SM and MM groups. We found increases in mRNA and protein expression of *shaker-related* voltage-gated K^+^ channels (Kv1: *Kcna1, Kcna3, and Kcna6*) and calcium-activated K^+^ channels (*Kcnn1*) in the SM compared to MM rats. SM rats also showed decreased DNA methylation at the CpG-rich sites near the promoter region of *Kcna1, Kcna3* and *Kcnn1* genes in comparison to MM rats. Together, these results provide additional evidence for potentially using K^+^ channels as therapeutic targets against METH use disorder.

**Electronic supplementary material:**

The online version of this article (10.1007/s12035-019-01830-3) contains supplementary material, which is available to authorized users.

## Introduction

METH use disorder (MUD) is a very serious, potentially lethal, biopsychosocial disease. For example, METH-related deaths rank 2nd after opioids in South East Asian countries and 3rd in most European nations [1]. According to the U.S. National Survey on Drug Use and Health [2], 375,000 Americans (aged 18–25) and 1.2 million (aged 26 or older) are active METH users. Despite the widespread use of METH, much remains to be done to develop effective therapeutic approaches to treat MUD [3, 4], a fact that is, in part, related to a relative lack of understanding of the molecular neurobiology of this brain disease.

Studies on potential molecular substrates of substance use disorders (SUDs) have identified some interesting targets for pharmacological interventions [5–7]. These include biochemical signaling pathways including the glutamatergic system [7], specific gene networks [8], as well as epigenetic regulatory mechanisms [6, 7]. Recent observations have also suggested that potassium (K^+^) channels might also be involved in modulating the compulsive intake of several drugs of abuse including alcohol [9, 10], cocaine [11], methamphetamine [5], nicotine [12], and opioids [13].

K^+^ channels regulate resting cell membrane potentials, modulate neurotransmitter release and neuronal excitability, as well as maintains cellular homeostasis [14–16]. The opening of these channels induces K^+^ efflux, hyperpolarization of cells, and decreases cellular excitability [14–16]. K^+^ channels are divided into subgroups based on their molecular structures and mechanisms of activation [17, 18]. These subgroups include (i) voltage-gated (Kv), (ii) inward rectifier (Kir), (iii) ion (calcium/sodium)-activated (KCa/Na), and (iv) two-pore (K2p) K^+^ channels. K_V_ channels are products of 40 genes in 12 subfamilies [19] whereas the related K_Ca_ channels are encoded by 8 genes in 4 subfamilies [20, 21]. Twenty-six K_V_ subunits are expressed in neuronal cells in the brain [22], with local translation of Kv channel mRNAs in neuronal processes leading to the diversification of axonal and dendritic action potentials [15]. Trafficking also plays significant roles in the sub-cellular localization of potassium channels in axon terminals [23] where they are involved in regulating various aspects of neurotransmission including dopamine (DA) neurotransmission in various brain regions including the nucleus accumbens (NAc) and midbrain DA neurons [24–26]. Indeed, the potential role for these channels in SUDs is supported by their localization and expression patterns in brain regions that regulate learning, reward, and addiction [27, 28]. These brain regions include the prefrontal cortex, hippocampus, NAc, and midbrain DA neurons [16, 29] that are key structures in the acquisition and/or maintenance of drug taking behaviors [30]. These channels are also found in brain regions that are involved in other cognitive functions such as decision making [31–33] that are relevant to addictive behaviors.

As mentioned above, there is substantial evidence to support various roles of K^+^ channels in the effects of psychostimulants. Specifically, mice treated with a single cocaine injection exhibited reduced GIRK current induced by stimulation with the GABAB agonist, baclofen, in VTA DA neurons [34]. Repeated injections of cocaine did not potentiate these changes [34]. These electrophysiological adaptations may be behaviorally significant since mice with more global deletion of the inward rectifying channel, Kir3/Girk3, were reported to exhibit decreased cocaine SA [35] whereas mice with Girk2 deletion in midbrain DA neurons showed increased cocaine intake in a SA paradigm [11]. Consistent with the latter findings, activation of the KCNQ2/3 channel by the agonist, flupirtine, significantly reduced cocaine conditioned place preference (CPP) in rats [36]. Moreover, acute [37] or repeated non-contingent injections of METH for 5 days [37, 38] caused decreases in the size of GABAB-activated GIRK currents in rodent VTA DA neurons. Of more direct relevance to the present study, METH SA also caused decreased GIRK currents in midbrain DA neurons of mice [39] while we recently found that suppression of METH SA behaviors in the presence of footshock punishment was associated with increased expression of K^+^ channels in the rat NAc [5].

In order to develop better therapeutic approaches to MUD, it is essential to elucidate the basic molecular processes that are responsible for the neuroadaptations that may be antecedent to the enduring behaviors observed in humans. Therefore, the main purpose of this study was to further investigate potential roles of K^+^ channels in the METH SA rat model. To reach that goal, we used a prior exposure of a single METH injection in an attempt to replicate enhanced drug SA reported in other studies exposed to psychostimulants including cocaine and amphetamine [40–45]. Based on the results of these previous studies, we hypothesized that METH pre-injected animals would show greater METH SA escalation compared to the saline-pretreated group. We also predicted that METH-pre-injected animals would show greater incubation of METH-seeking behaviors than rats pre-injected with saline before METH SA. Because K^+^ channels are key functional players in the regulation of diverse cellular functions including neurotransmitter release and neuronal excitability [17, 46, 47], we thought it likely that saline- and METH-pretreated animals would show differences in the expression of these channels, with rats pre-exposed to METH showing lower expression of K^+^ channels than the saline-pretreated animals and the controls based on the results of our previous study on the expression of potassium channels in METH SA rats [5]. We also sought to determine if changes in gene expression correlated with altered DNA methylation after METH SA. Using the METH pretreatment described here, we had previously identified differences in gene expression in the NAc of saline- and METH-pretreated rats [40] but had not tested the effects of that approach on METH SA.

## Results

### Intravenous METH SA Is Accentuated by a Single Prior Intraperitoneal METH Injection

**Fig. 1:**
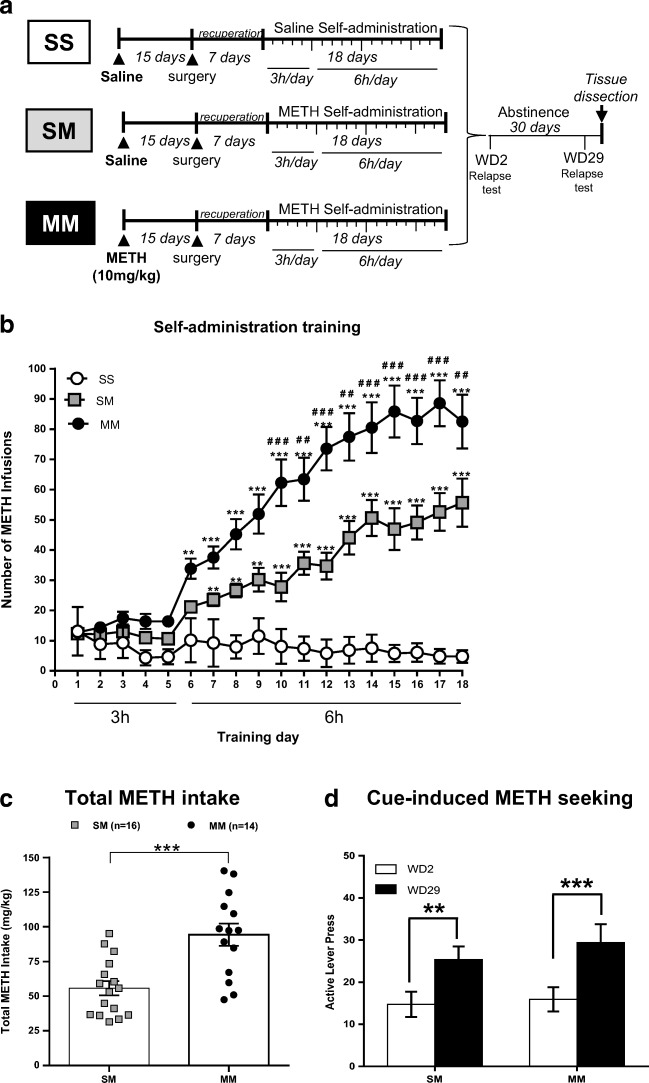
**(a)** Timeline of experimental events. Fifteen days after a single injection of saline or METH (10 mg/kg body weight) intravenous catheters were implanted. After additional 7 days of recuperating from surgery, rats were trained to lever press to self-administer saline or METH (0.1 mg./ infusion, delivered over 5 s). The rats were divided into (i) saline non-contingent injection followed by saline SA (SS, *n* = 12), (ii) saline non-contingent injection followed by METH SA (SM, *n* = 16) and (iii) METH-non-contingent injection followed by METH SA training (MM, *n* = 14). The number of hours that the animals had access to METH or saline are as shown in the figure. Cue-induced METH seeking tests were carried out on withdrawal days 2 (WD2) and 29 (WD29). The rats were euthanized and brain tissues were dissected 24 h after the second drug seeking tests. **(b)** Escalation of METH intake during METH SA training. Prior exposure to METH led to greater escalation of METH SA and of total drug taken during the period of training. During the first 5 days of training, there were no group differences in METH intake between SM and MM rats. Thereafter, both SM (*n* = 16) and MM (*n* = 14) rats escalated their METH intake, with MM rats showing significantly faster escalation compared to SM rats. Key to statistics: *P < 0.05, **P < 0.01, ***P < 0.001, in comparison to controls (SS); # *P* < 0.05, ## *P* < 0.01, ### *P* < 0.001, comparison between SM and MM rats. (**c**) Total amount of METH taken by rats from the SM (mean = 55.78 mg/kg) and MM (mean = 94.26 mg/kg) groups. Key to statistics: ***P < 0.001, comparison between SM and MM rats. (**d**) Incubation of METH seeking during withdrawal from METH SA. SM and MM rats showed similar incubation of METH craving during the month of withdrawal. Data are represented as means ± SEM of number of active lever presses. Key to statistics: **P < 0.01, ***P < 0.001 in comparison to WD2

The experimental design is illustrated in Fig. 1a. Rats were first given a non-contingent injection of either METH (10 mg/kg) or saline. Three weeks later, they underwent training for METH SA for a total of 18 days. There were three experimental groups in the SA experiments: (1) A control group consisting of rats that were injected with a saline injection followed by saline SA (SS, *n* = 12); (2) a second group consisting of rats given a single saline injection followed by METH SA (SM, *n* = 16); and (3) a third group consisting of rats that received a single METH injection followed by METH SA (MM, *n* = 14). Figure 1b shows the results of the behavioral SA experiment. As previously reported by our group [5, 48], rats that self-administer saline (SS) do not escalate their saline intake (Fig. 1b). The two METH groups did not show any significant escalation of METH intake during the first 5 days of training during which they had 3 h of access to METH. However, when given 6 h of access to METH, both SM and MM rats significantly escalated their METH intake. As predicted, the MM group showed greater escalation of METH intake than the SM group. Two-way ANOVA with repeated measures comparing SM and MM rats showed significant effects of group [F(_1, 28_) = 17. 01, *p* = 0.0003], training days [F(_17, 476_) = 65. 74, *p* < 0. 0001], and group × training days interaction [F(_17, 476_) = 6. 293, p < 0. 0001]. Figure 1c shows the total amount of METH taken by rats from the SM (mean = 55.78 mg/kg) and MM (mean = 94.26 mg/kg) groups (*p* < 0.001).

Unexpectedly, there were no significant differences in cue-induced METH seeking measured on days 2 (WD2) (white bars) and 29 (WD29) (black bars) after forced withdrawal from METH SA (Fig. 1d). Both groups showed similar incubation of METH craving at WD29 compared to WD2 (Fig. 1d). This is illustrated by two-way ANOVA with repeated measures comparing SM and MM groups showing significant effects of days [F(_1, 29_) = 26. 21, *p* < 0.0001], but no significant effects of group [F(_1, 29_) = 0. 395, *p* = 0.534] or group x withdrawal days interaction [F(_1, 29_) = 0. 365, *p* = 0.551].

Two-way ANOVA with repeated measures comparison for SM and MM groups body weight showed significant weight gain over the training days [F(_17, 544_) = 41. 56, p < 0.0001]; however, there were no significant effects of group [F(_1, 32_) = 0. 124, *p* = 0.912] over that time (Fig. S1).

### K^+^ Channel mRNA Levels Are Differentially Expressed between SM and MM Rats

Increased expression of two classes of K^+^ channels (Kv and Kca) has been observed in the NAc of rats that had significantly reduced their intake of METH in the presence of footshocks when these rats were compared to other rats that continued to take METH compulsively under similar conditions [5]. The persistent METH takers had taken more METH than the relatively abstinent ones [5]. To test the possibility that there might also be differences in the expression of K^+^ channels between the two groups that differed in their METH intake in the present study (see Fig. 1c above), we decided to measure mRNA levels for several members of these two families of K^+^ channels. We also measured the mRNA expression of these K^+^ channels in a group of rats that were treated with a single non-contingent METH injection (10 mg/kg dose) but had not been placed through the SA experiment (MS group). This group was used to measure the molecular effects of injection of METH alone. With the exception of *Kcnn2*, the K^+^ channels did not show any significant changes in their mRNA expression relative to the control (SS) group. These results are listed in the supplemental Table S2. For the sake of clarity, the figures shown below illustrate the results for the 3 groups that were involved in the SA experiments.

**Fig. 2:**
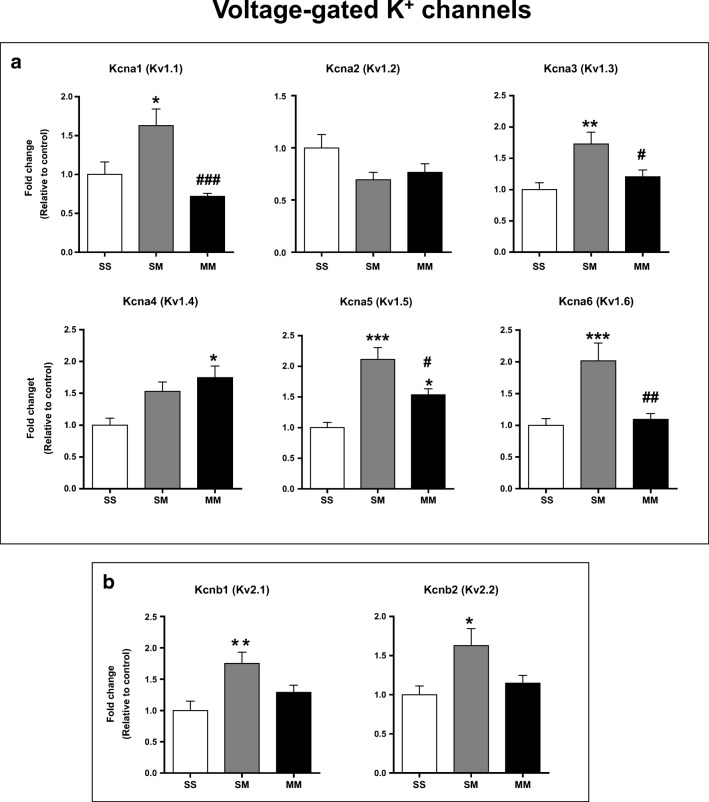
Effects of METH SA and abstinence on the expression of voltage-gated potassium (K^+^) channels in the NAc. (**a**) **Kv1** K^+^ channel mRNA levels. (**b**) **Kv2** K^+^ channel mRNA levels. Data are shown as means ± SEM of fold changes relative to the controls. Key to statistics: *P < 0.05, **P < 0.01, ***P < 0.001 in comparison to controls (SS group); # = *P* < 0.05, ## = *P* < 0.01, ### = *P* < 0.001 in comparison to SM rats

As shown in Fig. 2a, there were indeed significant differences in mRNA levels that code for Kv1 channels including *Kcna1* (*Kv1.1*) [F(_2, 23_) = 9.41, *p* = 0.001], *Kcna3* (*Kv1.3*) [F(_2, 23_) = 6.89, *p* = 0.0045], *Kcna5* (*Kv1.5*) [F(_2, 23_) = 17.32, *p* < 0.0001] and *Kcna6* (*Kv1.6*) [F(_2, 23_) = 9.631, *p* = 0.0011] between the NAc of SM rats and that of SS and MM rats. In contrast, *Kcna4* (*Kv1.4*) mRNA levels showed significant increases in the NAc of MM rats compared to the SS [F(_2, 23_) = 6.122, *p* = 0.0074] but not the SM rats (Fig. 2a). However, there were no significant differences in *Kcna2* (*Kv1.2*) expression between the groups (Fig. 2a).

Figure 2b illustrates the changes in gene expression for Kv2 voltage-gated channels. There were significant increases in *Kcnb1* (*Kv2.1*) [F(_2, 23_) = 6.32, *p* = 0.0065] and *Kcnb2* (*Kv2.2*) [F(_2, 23_) = 4.44, *p* = 0.023] mRNA levels in the SM group compared to the control group.

**Fig. 3:**
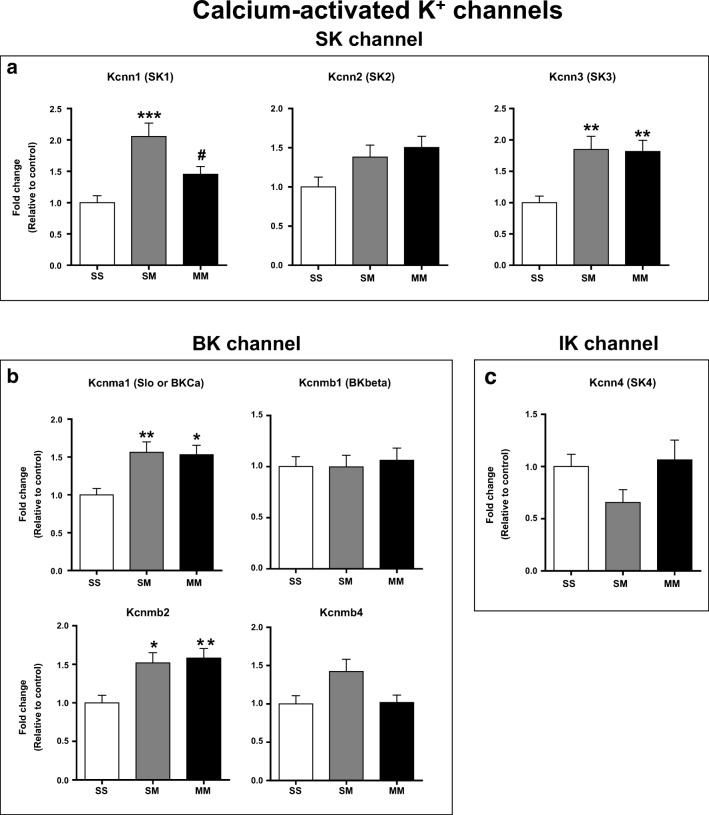
mRNA expression of calcium-activated K^+^ channels in the NAc of rats after 30 days of withdrawal from saline or METH SA. (**a**) *Small-conductance* (*SK*); (**b**) *Big-conductance* (*BK*); and (**c**) *Intermediate-conductance* (*IK*) calcium-activated K^+^ channels. Data are presented as means ± SEM of fold changes relative to the controls. Key to statistics: *P < 0.05, **P < 0.01, ***P < 0.001 in comparison to controls (SS group); # *P* < 0.05in comparison to SM rats

Changes in mRNA expression of small conductance (SK) calcium activated K^+^ channels are shown in Fig. 3a. *Kcnn1* (*SK1*) mRNA levels were significantly increased in SM rats compared to SS control and MM rats [F(_2, 23_) = 11.00, *p* = 0.0004]. METH SA did not impact the expression of *Kcnn2* (*SK2*) mRNA in either SM or MM rats (Fig. 3a). *Kcnn3* (*SK3*) mRNA levels were significantly increased in both SM and MM groups compared to controls [F(_2, 23_) = 7.30, *p* = 0.0035] (Fig. 3a). The expression of big conductance (BK) calcium activated K^+^ channels- *Kcnma1*(*BK*_*Ca*_ /*Slo*) [F(_2, 23_) = 6.66, *p* = 0.0052] and *Kcnmb2* [F(_2, 22_) = 7.04, *p* = 0.0043] were also significantly increased in both SM and MM groups compared to SS controls (Fig. 3b). These observations suggest that METH exposure alone is sufficient to increase the expression of Kcnn3, Kcnma1, and Kcnmb2. No significant differences were observed in mRNA levels for *Kcnmb1* (*BK*_*Beta*_), *Kcnmb2* (Fig. 3b) or *Kcnn4* (*SK4*) (Fig. 3c).

### METH SA Induces Differential Protein Expression of Voltage-Gated and Small-Conductance K^+^ Channels in the NAc

**Fig. 4:**
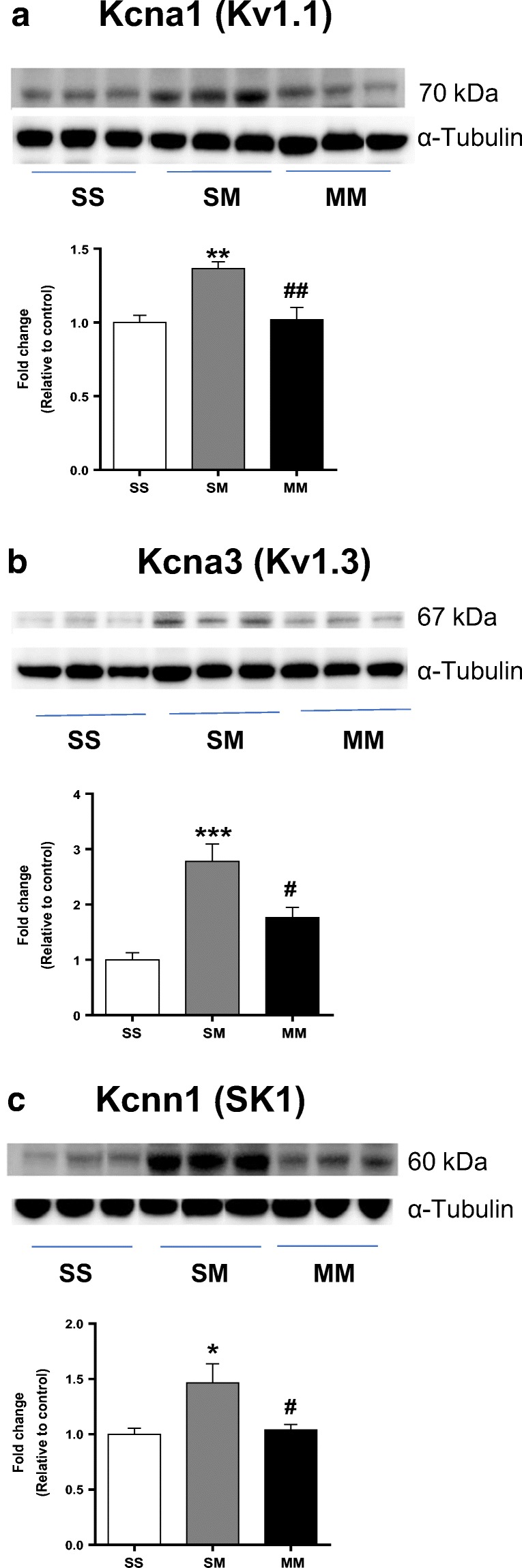
Increased protein levels of (**a**) KCNA1, (**b**) KCNA3, and (**c**) KCNN1 in the NAc of SM rats. Data are presented as means ± SEM of fold changes relative to the controls. Key to statistics: *P < 0.05, **P < 0.01, ***P < 0.001 in comparison to controls (SS group); # *P* < 0.05, ## *P* < 0.01 in comparison to SM rats

In order to test if changes in the expression of mRNAs of interest might also be reflected by changes in protein levels, we used Western blotting to measure levels of these proteins in membrane fractions from the NAc. Figure 4a and b illustrate significant increases in KCNA1 [F(_2, 12_) = 11.03, *p* = 0.0019] and KCNA3 [F(_2, 12_) = 15.83, *p* = 0.0004] protein levels, with SM rats having increased expression in comparison to both SS and MM rats. As shown in Fig. 4c, there were significant increases in KCNN1 protein levels [F(_2, 12_) = 5.787, *p* = 0.0174] in SM animals in comparison to MM (*P* < 0.05) and control (*P* < 0.05) groups**.** Thus, as predicted, the changes in protein levels reflected the changes in mRNA levels (compare to Figs. 2a and 3a).

### Changes in Gene Expression Are Associated with Altered DNA Methylation in the NAc of SM and MM Rats

**Fig. 5:**
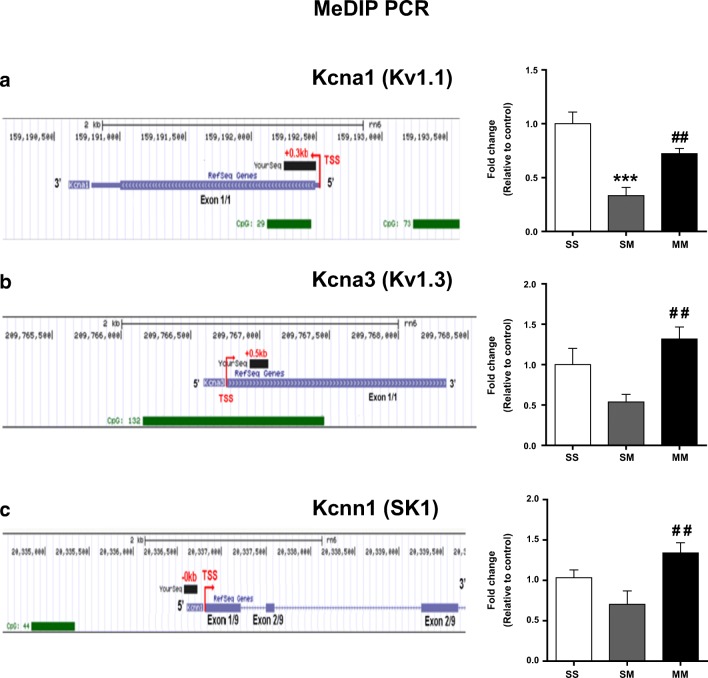
METH SA caused decreased DNA methylation in the NAc of SM rats. **(a)** DNA methylation at CpG-rich sites near the promoter of *Kcna1* voltage-gated K^+^ channels in the NAc of SM and MM rats. (**b**) DNA methylation at the *Kcna3* promoter showed a decreasing trend (*p* = 0.0548) in the NAc of SM rats in comparison to control rats; pre-exposure to METH normalized DNA methylation. (**c**) Decreased DNA methylation of calcium-activated K^+^ channel, *Kcnn1,* in the NAc of SM rats and this was also reversed in the MM rats. Key to statistics: ***P < 0.001 in comparison to controls (SS group); ## P < 0.01 in comparison to SM rats

In order to identify potential regulators of the differences in *Kcna1, Kcna3, Kcna5, Kcna6,* and *Kcnn1* mRNA levels observed between the SM and MM rats, we sought to determine if there were identifiable changes in DNA methylation at their promoter regions using MeDIP-qPCR assay. Gene expression has been shown to be partially dependent on changes in DNA methylation with increased and decreased mRNA levels resulting from DNA hypo- and hypermethylation, respectively [see review by Illingworth and Bird (2009) [49]]. We found that there was significant decreased DNA methylation at the *Kcna1* CpG-rich promoter region [F(_2, 20_) = 17.29, *p* < 0.0001] in SM rats in comparison to control and MM rats (Fig. 5a). Similarly, decreased DNA methylation was also observed at the *Kcna3* CpG-rich promoter region [F(_2, 23_) = 6.64, *p* = 0.0053] in SM rats in comparison to control and MM rats (Fig. 5b). *Kcna5* and *Kcna6* did not show any significant alterations in DNA methylation after exposure to METH (data not shown). We also detected significant DNA hypomethylation at the *Kcnn1* promoter region [F(_2, 22_) = 5.961, *p* = 0.0085] in the SM group in comparison to the MM group (Fig. 5c), a gene that also showed differential changes in gene expression.

## Discussion

The first aim of the present study was to measure the potential impact of a prior non-contingent injection of METH on METH SA. This paradigm is known to increase behavioral and/or molecular responses to psychostimulants [40–45]. The secondary aim was to assess the effects of METH SA on the mRNA levels of K^+^ channels in the NAc. The third aim was to test if there were changes in DNA methylation that could be offered as a partial explanation for the changes in mRNA expression. Our major findings are that *(i)* rats exposed to a single non-contingent dose of METH (10 mg/kg) or saline escalate their METH intake during SA; in addition, as hypothesized, the METH pre-injected animals showed greater METH SA escalation than the saline-pretreated group; *(ii)* unexpectedly, the SM and MM rats showed no differences in incubation of METH-seeking behaviors after four weeks of forced withdrawal from METH SA; *(iii)* the prior non-contingent injection of METH before METH SA was associated with attenuation of the effects of METH SA and withdrawal on mRNA and protein expression of *shaker-related* voltage-gated K^+^ channels (*Kcna1*/ *Kv1.1, Kcna3/Kv1.3, and Kcna6*/*Kv1.6*) and calcium-activated K^+^ channels (*Kcnn1*/*SK1*); and *(iv)* the prior injection of METH also attenuated METH SA-mediated decreased DNA methylation at the CpG-rich sites near the promoter regions of *Kcna1, Kcna3* and *Kcnn1* genes. The potential role of potassium channels in the behavioral differences observed between the two groups is discussed below. We also wrote about potassium channels as potential targets for treatment of SUDs.

### Differential METH SA and Incubation of METH Seeking

Previous reports have provided evidence that prior repeated exposure to psychostimulants can cause behavioral sensitization upon re-exposure to drugs [41, 50–52]. There is substantial evidence that animals repeatedly exposed to psychostimulants including cocaine and METH are more likely to self-administer smaller doses of psychostimulants than animals pre-exposed to saline [53–56]. On the other hand, very few studies have been conducted on the effects of single prior exposure to psychostimulants on subsequent drug-induced behaviors by the animals. It has been reported that a single pretreatment of amphetamine enhanced rotational behaviors induced by a second injection given 3–4 weeks later [57]. Similar observations were made for cocaine [58]. Interestingly, a single pretreatment with amphetamine caused sensitization to the locomotor effects of amphetamine that intensified over several weeks [44]. Consistent with the data reviewed above, we also found that rats pretreated with a single injection of showed greater escalation of METH SA and took larger amount of the drug than rats pretreated with saline. Unexpectedly, however, we found no significant differences in incubation between the two groups that differed in their METH intake. These observations suggest the existence of a ceiling that was reached by both groups once a certain amount of METH was self-administered. This idea will need to be tested in animals that are given different access to METH during drug SA experiments.

### METH Pretreatment, Enhancement of Escalated METH SA, and Potassium Channels

In order to dissect the molecular substrates of the METH pretreatment-induced enhancement of compulsive METH taking behaviors, we measured the impact of the pretreatment on any potential effects of METH SA on the mRNA expression of several K^+^ channels in the NAc. We chose that approach because we recently showed that rats that self-administered less METH had higher expression of some K^+^ channels in that structure [5]. Therefore, we reasoned that the SM group would have higher expression of potassium channels than the MM group since the SM rats took less METH than the MM rats. We also thought that the expression of these K^+^ channels might be lower than that of control rats. As expected, SM rats did show higher mRNA and protein expression of KCNA1 (Kv1.1) and KCNA3 (Kv1.3) in the NAc in comparison to the MM animals. There were, however, no differences between the MM group and the control. We also found that the SM rats showed decreased DNA methylation at the sequences of these genes. These molecular changes are very consistent with our previous report of higher expression of Kv1.1 mRNA level and increased DNA hydroxymethylation in the NAc of rats that had taken less METH than other rats that had continue to self-administer large amounts of METH even in the presence of footshock punishment [5]. In general, DNA methylation at the 5-methylcytosine in CpG contexts located in promoters has been implicated as a transcriptional repressor through the inhibition of the binding of transcriptional factors and through the recruitment of methyl binding proteins that can recruit additional proteins to form heterochromatin [59, 60]. Thus, the observed decreased in DNA methylation in the SM rats might be partially responsible for the increased *Kcna1* (*Kv1.1*) and *Kcna3* (*Kv1.3*) mRNA levels in those animals. The suggestion that the increased mRNA expression of *Kv1.1* and *Kv1.3* is secondary to decreased DNA methylation at the DNA sequences of these genes is consistent with the results of a previous study that had reported that increased DNA methylation at the Kv1.3 gene promoter region was associated with decreased *Kv1.3* mRNA levels in breast adenocarcinoma [61]. In any case, when taken together, it is not farfetched to suggest that increased expression of these K^+^ channels during METH SA might serve to keep METH taking behaviors in the maintenance phase of drug SA after initial escalation. However, it appears that a prior single injection of METH might have been sufficient not only to cause enhanced METH taking behavior but also to suppress the effects of METH SA on DNA methylation and changes in the expression of specific K^+^ channels.

In the present study, we also found that mRNA and protein levels of SK channel subunit-KCNN1 (SK1) were increased in the NAc of SM rats as compared to MM rats. These observations are of interest given that a recent database search of previous whole genome studies had suggested a role for KCNN1 in METH addiction [62]. KCNN1 may also be involved in other SUDs because activation of SK channels by chlorzoxazone can reduce excessive alcohol intake [63]. In addition, Mulholland et al. (2011) [64] have reported reduced SK channel function in the hippocampal CA1 region of rats that had undergone chronic ethanol exposure. Taken together with other publications related to alcohol use disorder [65], our findings indicate that SK channels may represent excellent targets for future studies in the field of SUDs.

### Potassium Channels as Therapeutic Targets for Substance Use Disorders

Experimental approaches to the treatment of METH use disorders have focused mainly on the monoamine hypothesis of addiction, with not a single pharmacological agent resulting in strong clinical improvement in patients who suffer from MUD [3]. Taken together with previous results on the effects of cocaine and METH on K^+^ channels [5, 34, 35], the present study suggests a role for K^+^ channels as potential targets for therapeutic interventions in psychostimulant use disorders. There is, at present, a wealth of knowledge about the various roles of K^+^ channels in the brain and periphery [66]. As mentioned above, these channels are localized in brain regions that play integral roles in learning, reward, and the development and maintenance of addictive states [16, 29]. In addition, there are several drugs that are known activators or inhibitors of K^+^ channels. Some of these agents that include chlorzoxazone, minoxidil, diazoxide, flupirtine, sulfonylureas are in clinical use in the US and/or Europe [65–67]. Given the lack of beneficial treatment for psychostimulant use disorders at this point in time, our results and those of others support the need to test potassium activators and/or inhibitors in preclinical models of cocaine and METH SA. Because some of these drugs are FDA-approved, it should be feasible to test the potential benefits of using these agents in clinical populations who have not responded to other pharmacological agents. It should be pointed out that K^+^ channels appear also to have additional roles in the abuse of non-psychostimulant drugs [68]. For example, Hopf et al. (2011) [63] have reported that the FDA-approved potassium channel activator, chlorzoxazone, reduced alcohol intake in rats. Pharmacological activation of the voltage- gated K_V_7 (Kcnq) with retigabine also reduced voluntary alcohol drinking by rodents [63, 69–71]. The data reviewed in this paper support the notion that activators and/or inhibitors of diverse K^+^ channels need to be tested in preclinical models of SUDs. Since some of these agents are already clinically available, it behooves clinical researchers to expand their approaches beyond the classical monoamine model of SUDs to test the potential benefits of these agents in patients who need better treatment.

### Summary

In conclusion, although we have provided supportive evidence that a non-contingent prior injection of METH can intensify escalation of METH SA and have observed changes in DNA methylation and gene expression that occur in rats with enhanced SA behaviors, our present results are, however, only correlative at this point. Therefore, additional studies are needed to test the role of each specific potassium channel gene in various stages of METH SA including escalation and maintenance phases as well as incubation of METH seeking during withdrawal. Nevertheless, when taken together with previous work demonstrating potential involvement of these channels in preclinical models of alcohol and cocaine use disorders, our results indicate important roles for K^+^ channels in SUDs in general. Finally, these results indicate that manipulations of specific K^+^ channels might represent important novel avenues for pharmacological interventions against MUD.

## Materials and Methods

### Animals and Drug Treatment

All animal treatments and procedures were approved by the National Institute of Drug Abuse Animal Care and Use Committee according to the *Guide for the Care and Use of Laboratory Animals* (ISBN 0–309–05377-3). Male Sprague-Dawley rats (Charles River Labs, Raleigh), weighing 250-300 g were housed in a humidity- and temperature-controlled (22.2 ± 0.2 °C) vivarium with free access to both food and water. All animals were allowed to acclimate to the facility for one week. Following habituation, rats were assigned to two groups of 10 rats and received a single intraperitoneal (i.p.) injection of either saline or METH (10 mg/kg) three weeks before undergoing METH SA training.

### Self-Administration Study

#### Intravenous Surgery

Two weeks after the non-contingent saline or METH injection, rats were anesthetized with an intraperitoneal injection of ketamine (50 mg/kg) and xylazine (5 mg/kg) for surgical insertion of polyurethane catheters (SAI Infusion Technologies, Lake Villa, IL) into the jugular vein as previously described [5]. The other end of the catheter was attached to an external back mount device that allowed for access to the catheter. These ports were closed with dust caps (Plastics One, Roanoke, VA). After surgery, rats were given subcutaneous injections of buprenorphine (0.1 mg/kg) to relieve pain. Rats were allowed one week to recover post-surgery. During recovery, and throughout the SA experiment, catheters were flushed every 48 h with 1 mL gentamicin (0.05 mg/ml, Henry Schein, Melville, NY) in sterile saline solution.

We selected to use the 3-week time-point to perform SA experiments after the non-contingent METH injection because of previous studies in which we had documented enhanced changes in gene expression in rats that were euthanized one month after a single injection of METH (10 mg/kg) [40]. Increased expression of stress-related genes was apparent after 2 weeks and remained elevated for one month [72].

#### SA Apparatus and SA Training

Rats were trained in SA chambers located inside sound-attenuated cabinets and controlled by a Med Associates Systems (Med Associates, St Albans, VT). Each chamber had two levers which were 8.5 cm above the grid floor. The catheters of rats in both METH SA and saline groups were attached to intravenous lines consisting of polyethylene50 tubing, protected by a metal coil, and connected to a liquid swivel (Instech Laboratories, Inc., Plymouth Meeting, PA, USA) allowing the rats free movement inside the SA chamber.

After the week of recovery following surgery, without any prior operant training, rats were given access on a fixed-ratio one (FR-1) schedule to METH according to our previously described protocol [73]. During the SA training segment of the study, which lasted 18 days, rats were housed in the SA chambers. Animals had free access to food and water that were available in water bottles and feeders hanging on the walls of all SA chambers. Each SA sessions began at the onset of the rat’s dark cycle, where the active and inactive levers were presented to the rats and a red house-light illuminated the chamber. The rats were trained to press for 0.1 mg/kg METH infusions under a fixed-ratio 1 with 20-s timeout reinforcement schedule. Pressing the active lever resulted in a flash of the house light, a tone, and an infusion of METH (0.1 mg/kg/infusion). Presses on the inactive lever resulted in no scheduled reinforcements. We trained the rats to self-administer METH in approximately four cycles, consisting of five days each, with a 2-day rest period between cycles. During each rest period, rats were disconnected from the intravenous lines and remained housed in the SA chambers. The 2-day rest period was used to prevent excessive weight loss known to occur when rats are given long access to the drug. The first 5 days consisted of one 3-h session whereas the rest of the SA experiment of 13 days duration consisted of two 3-h daily sessions separated by a 30-min interval of no access to the levers. During that interval, the house light was turned off. Control rats self-administered saline under the same conditions.

#### Forced Abstinence and Tests for Incubation

At the conclusion of SA training, rats were removed from the SA boxes and individually housed in their home cages with no access to METH. Intravenous catheters were covered using dust caps and the rats had access to food and water ad libitum. Cue-induced drug craving was then assessed at days 2 and 29 of withdrawal from the METH SA experiment. During both drug seeking test, rats were placed back in the SA boxes for a single 3 h session during which presses on the drug-associated lever resulted in the presentation of tone and light cues only, but no METH infusions were delivered. All rats tested on day 2 were also tested on day 29 of withdrawal. Animals were euthanized after the second drug seeking test.

### Tissue Collection

NAc tissues were dissected and immediately frozen on dry ice to be used in RT-qPCR, western blot analysis, and methylcytosine DNA immunoprecipitation (MeDIP) assays.

### Quantitative PCR Analysis of mRNA Levels

Total RNA was isolated from one NAc of one brain hemisphere using RNeasy Mini kit (Qiagen, Valencia, CA). Unpooled total RNA (0.5 μg) isolated from NAc samples was reverse-transcribed with oligo dT primers using Advantage RT-for-PCR kit (Clontech, Mountain View, CA). RT-qPCR was performed essentially as described previously [74] with Roche LightCycler 480 II (Roche Diagnostics Corp., Indianapolis, IN) using iQ SYBR Green Supermix (Bio-Rad, Hercules, CA). For all RT-qPCR experiments, individual data were normalized using the corresponding 18S mRNA level. The results are reported as fold changes calculated as the ratios of normalized gene expression data for METH-treated groups (at various time-points) in comparison to the control group (SS). Primers for were synthesized at the Synthesis and Sequencing Facility of Johns Hopkins University (Baltimore, MD) and are listed in Supplemental Table S1.

### Immunoblot Analysis

Western blot analyses were carried out from NAc protein lysates (*n* = 6). Samples were homogenized separately in 10% *w*/*v* of ice-cold 10 mM HEPES buffer (pH 7.4) containing 10 mM KCl, 1.5 mM MgCl_2,_ 1%-Igepal CA-630 supplemented with a Roche protease inhibitor cocktail tablet (Roche Diagnostics). The homogenate was centrifuged for 5 min at 14,000 x g to pellet nuclear fraction. The supernatant was considered to be the cytosolic fraction. Protein concentrations of the cytosolic fractions were determined by a BCA assay (Thermo Fisher Scientific) and were then denatured with sample buffer (62.5 mM Tris-HCl, 10% glycerol, 2% SDS, 0.1% bromophenol blue, and 50 mM DTT) at 100 °C for 5 min, and then subjected to SDS-PAGE. Proteins were electrophoretically transferred to Hybond-PVDF membrane (Amersham). Subsequently, the membranes were incubated overnight at 4 °C with specific antibodies against KCNA1(Abcam, catalog # ab32433), KCNA3 (Santa Cruz, catalog # sc-398,855) and KCNN1 (Abcam, catalog # ab66624). After incubation, the blots were washed with TBS containing 0.1% Tween-20. The membranes were then incubated with HRP-conjugated anti-rabbit, anti-mouse or anti-goat secondary antibody for 1 h at room temperature. To confirm equal protein loading, the blots were re-probed with α-tubulin antibody (1:4000, 2 h at room temperature; Sigma). ECL plus chemiluminescent reagents (GE Healthcare) were used to detect protein expression. Signal intensity was measured with Carestream Molecular Imaging software. All experiments were duplicated.

### Methylated DNA Immunoprecipitation (MeDIP)

Genomic DNA was isolated from NAc tissues by overnight Proteinase K treatment, phenol-chloroform extraction, ethanol precipitation, and RNase digestion as previously described by Jayanthi et al. (2017) [72]. Subsequently, 300 μL fractions of DNA (20 μg) were sheared by ultrasonic treatment using the Diagenode Bioruptor (12 cycles, 30 s “ON”, 30 s “OFF”) to obtain a fragment size between ~200–600 bp. After denaturation (10 min at 95 °C), 5 μg DNA was then immunoprecipitated overnight at 4 °C using 5 μL of mouse monoclonal anti-5mC antibody (Millipore) for MeDIP assay in a final volume of 500 μL IP buffer (10 mM sodium phosphate (pH 7.0), 140 mM NaCl, 0.05% Triton X-100). We incubated the mixture with 80 μL of Dynabeads (Life Technologies) overnight at 4 °C and washed it three times with 700 μL of IP buffer. We then treated the beads with proteinase K for 3 h at 50 °C and recovered the methylated or hydroxymethylated DNA by phenol-chloroform extraction followed by ethanol precipitation. Sheared “input” DNA samples were collected prior to immunoprecipitation for subsequent comparison with immunoprecipitated DNA.

#### qPCR on MeDIP Samples

We carried out qPCR reactions with 20 ng of input DNA and immunoprecipitated methylated DNA. For qPCR reactions, we used the iQ SYBR Green PCR master mix (BioRad) and Roche 480 thermal cycler (Roche Diagnostics). The primer sequences are shown in Table S1. Reactions were done in duplicates and standard curves were calculated on serial dilutions (100–0.1 ng) of input genomic DNA. To evaluate the relative enrichment of target sequences after MeDIP, we calculated the ratios of the signals in the immunoprecipitated DNA versus input DNA.

### Statistical Analysis

Graph Pad Prism (v6,GraphPad software, SanDiego, CA) was used to statistically analyze datasets and create graphs. For SA experiment analysis, two-way repeated-measures ANOVA followed by Dunnett’s post-hoc tests were used with SA training days as the within-subjects factor, and experimental group as the between-subjects factor. For qPCR, MeDIP and western blot quantitative data, one-way ANOVA followed by Tukey’s post hoc analysis was used.. All the quantitative data are presented as mean + SEM. For all experiments, the null hypothesis was rejected at *p* < 0.05.

## Electronic supplementary material

ESM 1 (PPTX 85 kb)

ESM 2 (DOCX 16.5 kb)

ESM 3 (DOCX 16 kb)
