# Supplementary material for: A Single Prior Injection of Methamphetamine Enhances Methamphetamine Self-Administration (SA) and Blocks SA-Induced Changes in DNA Methylation and mRNA Expression of Potassium Channels in the Rat Nucleus Accumbens
Source: Mol Neurobiol. 2019 Nov 22;57(3):1459–72. doi: 10.1007/s12035-019-01830-3 (PMC7060962; doi:10.1007/s12035-019-01830-3)
Supplement: Supplementary file 1 — (PPTX 85 kb) [file 12035_2019_1830_MOESM1_ESM.pptx]

## Slide 1
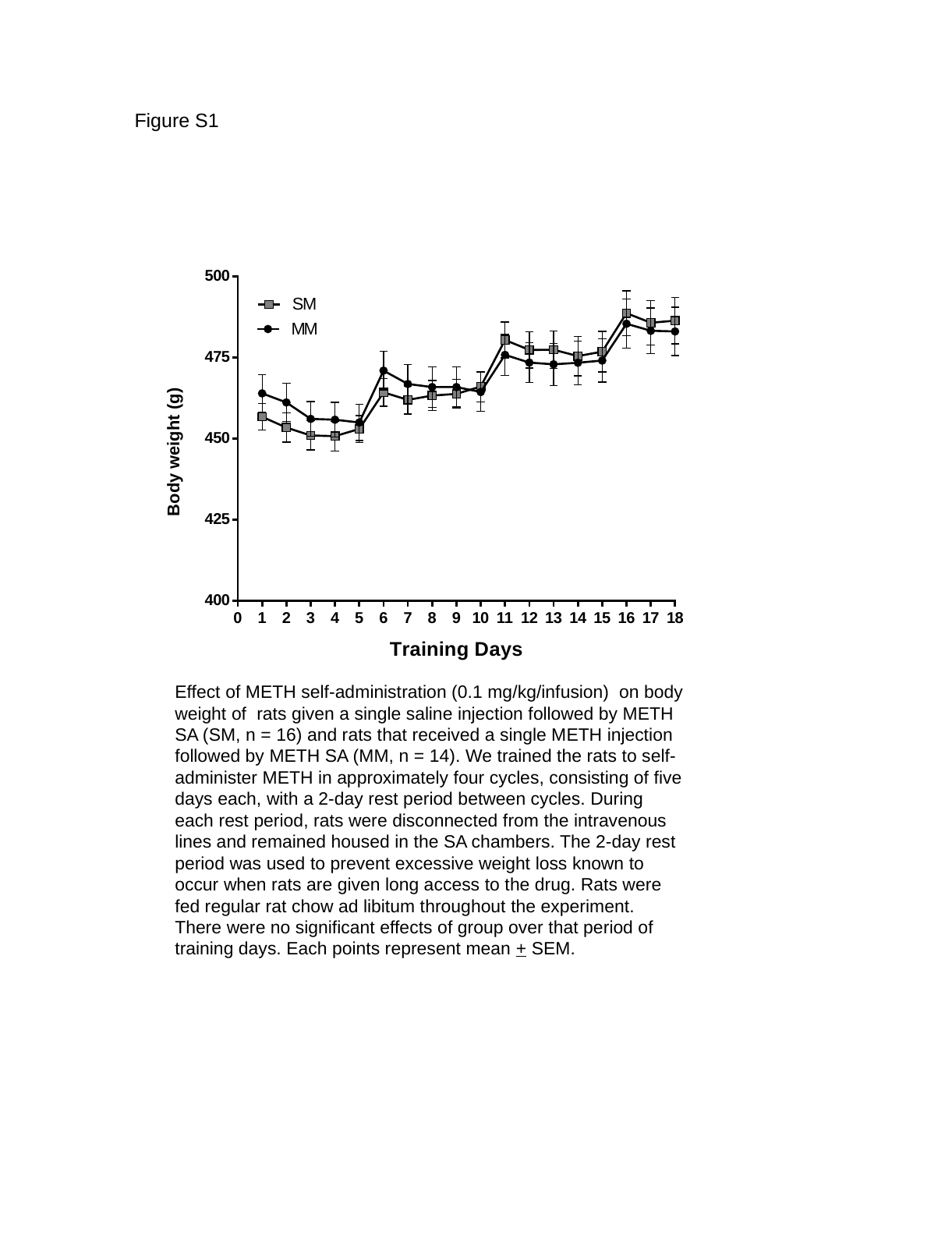

Figure S1
Effect of METH self-administration (0.1 mg/kg/infusion) on body weight of rats given a single saline injection followed by METH SA (SM, n = 16) and rats that received a single METH injection followed by METH SA (MM, n = 14). We trained the rats to self-administer METH in approximately four cycles, consisting of five days each, with a 2-day rest period between cycles. During each rest period, rats were disconnected from the intravenous lines and remained housed in the SA chambers. The 2-day rest period was used to prevent excessive weight loss known to occur when rats are given long access to the drug. Rats were fed regular rat chow ad libitum throughout the experiment. There were no significant effects of group over that period of training days. Each points represent mean + SEM.
