# Supplementary material for: A Single Prior Injection of Methamphetamine Enhances Methamphetamine Self-Administration (SA) and Blocks SA-Induced Changes in DNA Methylation and mRNA Expression of Potassium Channels in the Rat Nucleus Accumbens
Source: Mol Neurobiol. 2019 Nov 22;57(3):1459–72. doi: 10.1007/s12035-019-01830-3 (PMC7060962; doi:10.1007/s12035-019-01830-3)
Supplement: Supplementary file 2 — (DOCX 16.5 kb) [file 12035_2019_1830_MOESM2_ESM.docx]

**Table S1. RT-PCR and MeDIP PCR primers sequences**

| **Gene** | **Experiment** | **Forward primer (5’ to 3’)** | **Reverse primer (5’ to 3’)** |
| --- | --- | --- | --- |
| ***Kcna1*** | qRT-PCR | TTGGTAAGGGTGTTCAGAAT | GCAAAGTACACTGCACTAGA |
|  | MeDIP-PCR | AGTACTCATTCCTCAGAGGGTCA | ATCCATCATGACGGTGATGTCAG |
| ***Kcna2*** | qRT-PCR | GTGAGAGAGTGGTGATTAAC | TATCTAAGGGCACATTCACAG |
| ***Kcna3*** | qRT-PCR | TCATCTTCTGCTTGGAGACA | TATTTCTGGAGAAGGTGGCTTTA |
|  | MeDIP-PCR | CGCGGTTCTAATAGCCCTGAGAT | AGACTGGCCTTGGAGGAACC |
| ***Kcna4*** | qRT-PCR | CATGACAACTGTGGGCTACGG | CGGGCAAAGCAATGGTTAAGA |
| ***Kcna5*** | qRT-PCR | TTCTCTAGTATCCCAGATGC | CCCGATGATAGAAGTAATTAAAG |
| ***Kcna6*** | qRT-PCR | TATGGAAGAGATTCGCTTCTA | GAACTCTCCGGATACTCAAA |
| ***Kcnb1*** | qRT-PCR | CTCCATCTACACCACAGCAAGT | CTGAACTTGGGACTGGTACTCC |
| ***Kcnb2*** | qRT-PCR | GAAGAACTTAGAAGGGAGGC | GATGAACAGGATAGACACGAT |
| ***Kcnn1*** | qRT-PCR | GGCTCATCTCCATTACC | CGTTTTTAACCCGCTT |
|  | MeDIP-PCR | GGTGTCTGGAAGGTAGAGATAGC | AGACAAGAAAGAGCCATCACTGT |
| ***Kcnn2*** | qRT-PCR | TATGCGCTCATCTTCGGCAT | ACTGTATTTCCCTGGCGTGG |
| ***Kcnn3*** | qRT-PCR | GGTGATAGAGACCGAAC | TCAGGTATAGGCGCAA |
| ***Kcnn4*** | qRT-PCR | GGTTAAGTGTTTAATCACGC | ATCGGACTTGGTTGAG |
| ***Kcnma1*** | qRT-PCR | ACTCGTGAACGATACTAA | CTGTAACCCCCTCGAA |
| ***Kcnmb1*** | qRT-PCR | GGAATCCACCTGTCACT | GGTGTTTGTAGAAATTGGCT |
| ***Kcnmb2*** | qRT-PCR | ATCTTACAGACACGACGAG | GCATGTACGAGCGCAG |
| ***Kcnmb4*** | qRT-PCR | CTGACTAACCCCAAGTG | ATCTCGTCATGTGTGC |
