# Supplementary material for: A Single Prior Injection of Methamphetamine Enhances Methamphetamine Self-Administration (SA) and Blocks SA-Induced Changes in DNA Methylation and mRNA Expression of Potassium Channels in the Rat Nucleus Accumbens
Source: Mol Neurobiol. 2019 Nov 22;57(3):1459–72. doi: 10.1007/s12035-019-01830-3 (PMC7060962; doi:10.1007/s12035-019-01830-3)
Supplement: Supplementary file 3 — (DOCX 16 kb) [file 12035_2019_1830_MOESM3_ESM.docx]

**Table S2. Fold change of mRNA expression in MS group**

| **Gene** | **Fold change relative to SS group** | **P-value** |
| --- | --- | --- |
| *Kcna1(Kv1.1)* | 0.738 + 0.19 | ns |
| *Kcna2 (Kv1.2)* | 0.683 + 0.13 | ns |
| *Kcna3 (Kv1.3)* | 1.104 + 0.19 | ns |
| *Kcna4 (Kv1.4)* | 1.540 + 0.23 | ns |
| *Kcna5(Kv1.5)* | 1.396 + 0.18 | ns |
| *Kcna6 (Kv1.6)* | 1.037 + 0.15 | ns |
| *Kcnb1 (Kv2.1)* | 1.110 + 0.20 | ns |
| *Kcnb2 (Kv2.2)* | 1.077 + 0.19 | ns |
| *Kcnn1(SK1)* | 1.289 + 0.21 | ns |
| *Kcnn2 (SK2)* | 2.860 + 0.28 | p < 0.001 |
| *Kcnn3 (SK3)* | 1.266 + 0.23 | ns |
| *Kcnn4 (SK4)* | 0.774 + 0.18 | ns |
| *Kcnma1 (Slo / BKCa)* | 1.401 + 0.17 | ns |
| *Kcnmb1 (BK beta)* | 1.008 + 0.15 | ns |
| *Kcnmb2* | 1.436 + 0.17 | ns |
| *Kcnmb4* | 0.905 + 0.16 | ns |

Transcript levels of K^+^ channel genes were measured from the NAc of individual rats from the MS group (n = 9). Values are mean fold changes relative to the SS group. Key to statistics: ***p<0.001, in comparison to the SS group;
